# Effect of Deep Frying of Potatoes and Tofu on Thermo-Oxidative Changes of Cold Pressed Rapeseed Oil, Cold Pressed High Oleic Rapeseed Oil and Palm Olein

**DOI:** 10.3390/antiox10101637

**Published:** 2021-10-18

**Authors:** Małgorzata Wroniak, Marianna Raczyk, Bartosz Kruszewski, Edyta Symoniuk, Dominika Dach

**Affiliations:** 1 Department of Food Technology and Assessment, Division of Fats & Oils and Food Concentrates Technology, Institute of Food Sciences, Warsaw University of Life Sciences—SGGW, Nowoursynowska 159 C, 02-776 Warsaw, Poland; malgorzata_wroniak@sggw.edu.pl (M.W.); edyta_symoniuk@sggw.edu.pl (E.S.); dach.dominika@gmail.com (D.D.); 2 Department of Food Technology and Assessment, Division of Fruit, Vegetable and Cereal Technology, Institute of Food Sciences, Warsaw University of Life Sciences—SGGW, Nowoursynowska 159 C, 02-776 Warsaw, Poland

**Keywords:** frying, cold pressed rapeseed oil, high oleic rapeseed oil, palm olein, oxidative stability, fatty acids, Rancimat test, chlorophylls, carotenoids, oil quality

## Abstract

One of the commonly used food preparation methods is frying. Fried food is admired by consumers due to its unique taste and texture. Deep frying is a process of dipping food in oil at high temperature, usually 170–190 °C, and it requires a relatively short time. The aim of this study was to analyze the thermo-oxidative changes occurring during the deep frying of products such as potatoes and tofu in cold pressed rapeseed oils and palm olein. Cold pressed rapeseed oil from hulled seeds (RO), cold pressed high oleic rapeseed oil from hulled seeds (HORO), and palm olein (PO) (for purposes of comparison) were used. Characterization of fresh oils (after purchase) and oils after 6, 12, and 18 h of deep frying process of a starch product (potatoes) and a protein product (tofu) was performed. The quality of oils was analyzed by determining peroxide value, acid value, p-anisidine value, content of carotenoid and chlorophyll pigments, polar compounds, smoke point, color (CIE L*a*b*), fatty acids content and profile, calculation of lipid nutritional quality indicators, and oxidative stability index (Rancimat). Cold pressed high oleic rapeseed oil was more stable during deep frying compared to cold pressed rapeseed oil, but much less stable than palm olein. In addition, more thermo-oxidative changes occurred in the tested oils when deep frying the starch product (potatoes) compared to the deep frying of the protein product (tofu).

## 1. Introduction

Rapeseed is widely cultivated worldwide for the production of animal feed, vegetable oil, and biodiesel. The main producers of rapeseed oil are the European Union (EU), Canada, China, and India. Fat content of rapeseeds is around 40–45%. Rapeseed has a predominant content of oleic acid (57–63%), a high content of polyunsaturated fatty acids (30%), a low content of saturated fatty acids (6–7%), and an optimal nutritional ratio of n − 6:n − 3 acids (3:1 or 2:1). Additionally, it contains bioactive compounds such as tocopherols and phytosterols [1]. In recent years, high oleic rapeseed varieties have become more popular in food processing. Those varieties have higher oxidative stability due to the lower content of polyunsaturated fatty acids, in favor of a higher content of oleic acid [2,3]. Rapeseed oil can be obtained by cold pressing, but it is usually subjected to refining processes for obtaining a higher performance and repeatable quality [4].

The palm oil is one of the most consumed oils worldwide. The main producers of palm oil are Indonesia, Malaysia, Thailand, Colombia, and Nigeria [5]. On the other hand, the regions with the highest demand for crude palm oil include China, India, and the EU [6]. In contrast to rapeseed oil, palm oil has a much higher content of saturated fatty acids. Palm olein contains 38.3% of palmitic acid, 42.1% of oleic acid, and 10.6% of linoleic acid. It is more stable than vegetable oils containing a significant amount of polyunsaturated fatty acids. Hence, it seems reasonable to use this oil for the production of functional and convenient food. However, the most important reason for the wide range of applications and the diverse use of palm oil is its relatively low price [7].

One of the methods for food preparation used worldwide is frying. Deep frying is a process of dipping food in oil at a temperature between 130 and 190 °C. In the industry, different kinds of plant oils are used as a deep frying medium, mostly in mixtures [5]. High contents of saturated fatty acids and antioxidants are the basis for their better stability [8]. Frying oils should be easily available, inexpensive, stable during storage and processing, and of acceptable taste. Among the most popular oils used for frying are palm oil and its fractions, sunflower oil (especially high-oleic sunflower oil), rapeseed (canola), and soybean oils. For instance, palm olein is cheaper than rapeseed oil, but it has a higher saturated fatty acids content and less antioxidants; thus, alternatives with a more balanced fatty acids composition, more tocopherols, and more carotenoids are demanded [7]. However, during the heating process, oil undergoes thermal and oxidative changes, resulting in the formation of by-products, which affects its nutritional and sensory properties. The selection of a frying medium is very important, since a high amount of the oil is absorbed during the process, in some cases reaching 40% of the total food product weight [9]. Still, the amount of absorbed oil may vary significantly, as it is affected by many factors such as moisture content, fried food geometry, crust microstructure, frying oil temperature, frying time, and type of oil [5,7].

The oil oxidation at a high temperature that occurs during deep frying leads to the formation of degradation products such as triacylglycerol polymers, triacylglycerol dimers, oxidized triacylglycerol monomers, diacylglycerols, and free fatty acids [8]. An oil which is intended to be used as a frying medium should fulfill many requirements. First of all, a good fatty acids composition, which means the lowest possible content of fatty acids in the *trans* configuration (<1%), low content of saturated fatty acids (<30%), and low content of polyunsaturated acids—linoleic and linolenic (<2%) [10,11]. Additionally, it should have a high resistance to oxidation—high smoke point and high content of antioxidants [12,13]. Therefore, in this study we compared three different oils in the deep frying process of protein and starch products [5,6,10].

Cold pressed oils are mainly intended to be used cold, e.g., in dressings and spreads. However, a high oleic rapeseed oil could be used in cooking and frying, due to the high content of oleic acid, similar to that of olive oil [14]. There is a lack of data on frying products immersed in cold pressed high oleic rapeseed oil. Also, the effect of a high protein food like tofu on oil parameters during deep frying is little. Thus, it has been considered reasonable to evaluate the thermo-oxidative changes of cold pressed rapeseed oil, cold pressed high oleic rapeseed oil, and palm olein during the deep frying of starch and protein products.

## 2. Materials and Methods

### 2.1. Chemicals

All of the reagents and solvents used, of the HPLC/GC purity, were purchased from Merck (Darmstadt, Germany). The Supelco 37 Component Fatty Acid Methyl Esters (FAME) mix standard was supplied by Sigma-Aldrich Chemical Co., (St. Louis, MO, USA). Distilled water (0.05 mS) was obtained with an HLP Smart 2000 apparatus (Hydrolab, Poland).

### 2.2. Research Material

Cold pressed rapeseed oil (RO) (Złoto Polskie, Kalisz, Poland), cold pressed high oleic rapeseed oil (HORO) (Złoto Polskie, Kalisz, Poland), and palm olein (PO) (Salangor, Malaysia) were used in the study. Both types of rapeseed oil were obtained by cold pressing of hulled seeds. As fried materials, natural tofu (pieces of 5 × 1 × 1 cm) and local potatoes of Irga variety (pieces of 5 × 1 × 1 cm) purchased in a retail store in Warsaw, Poland, were used.

Experiments were repeated three times. Each repetition was carried out for a total of 18 h at 180 ± 5 °C in a SilverCrest fryer SFM 850 A5 (Hamburg, Germany). The initial oil amount of each experiment was 1.1 L. Every hour, a new 100 ± 2 g portion of tofu or potatoes was fried in separated fryers until reaching a golden-brown color. Tofu was fried for 7 min and potatoes for 8 min. Between frying each portion of material, the oil was kept at 180 °C. Oil samples for testing were taken before frying and after 6, 12 and 18 h of the experiment, cooled and kept in 200 mL glass jars and stored frozen (−20 °C) until the analyses.

### 2.3. Acid Value, Peroxide Value and p-Anisidine Value

Hydrolytic changes of the tested oils were specified by a titration method as the acid values (AV) according to the AOCS Official Method Cd 3d-63 [15]. The peroxide value (PV) of the oils was determined using a titration method according to the AOAC Official Method 965.33 [16]. The degree of oil oxidation based on the content of secondary oxidation products, particularly aldehydes, was given as *p*-anisidine values (AnV) based on the AOCS Official Method Cd 18–90 [17]. Spectrophotometric measurements for AnV were taken with a quartz cuvette with the 10 mm optical path length on Helios Gamma UV-Vis Spectrophotometer (Loughborough, UK).

### 2.4. Chlorophyll, Carotenoid Pigments and Color Determination

Both chlorophyll and carotenoid pigments were determined in triplicate for each sample by measuring absorbance at specific wavelengths using Helios Gamma UV-Vis Spectrophotometer (Loughborough, UK) with an optical glass cuvette and applying the 10 mm optical path length.

Chlorophyll pigments were determined according to Raczyk et al. [18] using the AOCS Official Method Cc 13i-96 with absorbance measurements at the wavelengths 630, 670 and 710 nm [19]. Quantity of pigments was expressed as mg pheophytin/kg oil.

Carotenoid pigments were assayed according to Rękas et al. [20] with the method described by BS 684-2.20 analytical standard [21]. Briefly, an oil sample was weighed into a 10 or 25 mL volumetric flask and filled with isooctane (the concentration of the solution was chosen appropriately to obtain an absorbance value in the range of 0.2 to 0.8). The absorbance was measured at the wavelength 446 nm against isooctane. Quantity of pigments was expressed as mg β-carotene/kg oil.

The color of the oil samples was measured with a colorimeter Konica Minolta CM-3600d (Tokyo, Japan) in CIE L*a*b* scale, calibrated against black and white plate standards. The parameters of the device in the transmission mode were set as follows: a standard observer of 10°, and an illuminant D65. L* (lightness), and b* (yellow to blue) values were measured using a glass cuvette (0.2 cm path) at room temperature.

### 2.5. Smoke Point

Smoke point was determined according to the AOCS Official Method Cc 9a-48 using a SYD-3536 Cleveland Open Cup Point Tester (Koehler, New York, NY, USA) [22]. Briefly, an oil sample was poured into the measuring cup and then heated rapidly to half the estimated smoke point, then the temperature was increased by 5 °C per minute. The measurement of the oil samples was done before frying and after 18 h of use, in triplicate.

### 2.6. FAMEs

Preparation of fatty acid methyl esters was done according to the AOCS Official Method Ce 2–66 [23]. Fatty acid composition of each oil sample of the methyl esters obtained by transmethylation of the oil with KOH in methanol was determined using TRACE™ 1300 gas chromatograph (Thermo Scientific, Waltham, MA, USA), equipped with FID detector and BPX 70 capillary column (60 m length, 0.22 mm i.d., 0.25 mm film thickness). Helium was used as a carrier gas at a flow rate of 0.5 mL min^−1^, the injection volume was 1 µL at a split ratio of 500:1. The GC’s oven temperature was programmed as follows: 80 °C hold for 2 min, ramped to 230 °C at a rate of 2.5 °C min^−1^, hold for 6 min. Identification of FAMEs was performed by comparing peaks retention times with those of reference standards (mixture FAME Mix, Supelco, which included 37 FAMEs). For quantification of FAMEs, methyl-nonadecanoate (Sigma Aldrich Chemical Co., St. Louis, MO, USA) was used as the internal standard.

### 2.7. Rancimat Test

Oxidative stability of the oils was determined using a 743 Rancimat Metrohm apparatus (Herisau, Switzerland) according to the AOCS method Cd 12b-92 [24]. The test was carried out at a constant temperature at 120 °C with air flow of 20 L/h, using 2.5 g oil samples and 0.06 L distilled water in a conductometric vessel. The result of measurements was a curve with automatically appointed induction time which was calculated by the apparatus software. The oxidation stability index (OSI) was expressed in hours (h).

### 2.8. Total Polar Compounds

The content of total polar compounds (TPC) was determined using the Testo 270 tester (Lenzkirch, Germany) according to the manufacturer’s instructions. A sample of about 50 mL was heated to a temperature of 40 °C and then the measuring part of device was immersed in the oil. The content of the TPC was expressed as a percentage.

### 2.9. Oxidizability (COX) Value and Nutritional Quality Indices of Oils

In order to determine what effect the fatty acid composition of oils has on the risk of developing cardiovascular disease, lipid quality indices have been determined. In order to calculate them, it is necessary to know the fatty acids profile of the fats/oils under the study. The oxidizability (COX) value was calculated based on the content of unsaturated C18 fatty acids, applying the Equation (1) proposed by Fatemi and Hammond [25].

COX = [C18:1 + (10.3 ∗ C18:2) + (21.6 ∗ C18:3)]/100(1)

The atherogenicity index (AI) assesses the likelihood of development of atherosclerosis. It is expressed by the ratio of selected saturated acids to unsaturated acids, and calculated by the Equation (2). The thrombogenicity index (TI) is designed to determine the prothrombotic effect of fat consumed as part of the diet. With this index, it is possible to assess the affinity level of platelets to clump together in blood vessels. The TI index was computed using the Equation (3). Both the AI and the TI index were evaluated following the calculations proposed by Ulbricht and Southgate [26].

AI = [C12:0 + (4 ∗ C14:0) + C16:0]/[ΣMUFA + Σ(n − 3) + Σ(n − 6)](2)

TI = [C14:0 + C16:0 + C18:0]/[(0.5 ∗ ΣMUFA) + (0.5 ∗ Σ(n − 6)) + (3 ∗ Σ(n − 3)) + (n − 3/n − 6)](3)

The cholesterolaemic effect of the fat source can be expressed as the ratio of hypocholesterolaemic acids (DFA) to hypercholesterolaemic acids (OFA) (named as hH ratio). The function of DFA acids (unsaturated C18, C20 fatty acids) is to lower the level of total cholesterol in the blood, while OFA acids (C14:0, C16:0) increase this level. The hH ratio was calculated based on the Equation (4) proposed by Santos-Silva et al. [27].

hH = (C18:1 + C18:2 + C18:3 + C18:4 + C20:4)/(C14:0 + C16:0)(4)

### 2.10. Calculations and Statistics

The statistical program Statistica 13.3 (TIBCO Software Inc., CA, USA) was used to develop the results. The results were obtained using one-way analysis of variance (ANOVA). In order to evaluate the differences between average values for data that was normally distributed, the Tukey HSD test was used at a significance level of *α* = 0.05. If the tested data did not come from normal distribution, the Kruskal–Wallis test was used instead.

The gathered quantitative data were used in the chemometric analysis in principal component analysis (PCA) in order to show the level of thermo-oxidative changes in oil samples after 18 h of frying, as well as to present the affinity of oils between samples used for frying different matrix composition food products. The results of the determinations performed were qualified for PCA analysis based on a correlation score with the first or second principal component at a level of at least 0.7 [28]. According to the generated factor loadings matrix, data from the following analyses of oils were qualified for PCA classification: acid, peroxide, *p*-anisidine values, TPC, chlorophyll and carotenoid pigments content, L* and b* color values, smoke point, OSI value, ΣSFA, ΣMUFA, ΣPUFA, n − 6/n − 3 ratio.

## 3. Results and Discussion

### 3.1. Acid Value, Peroxide Value, and p-Anisidine Value

**Figure 1:**
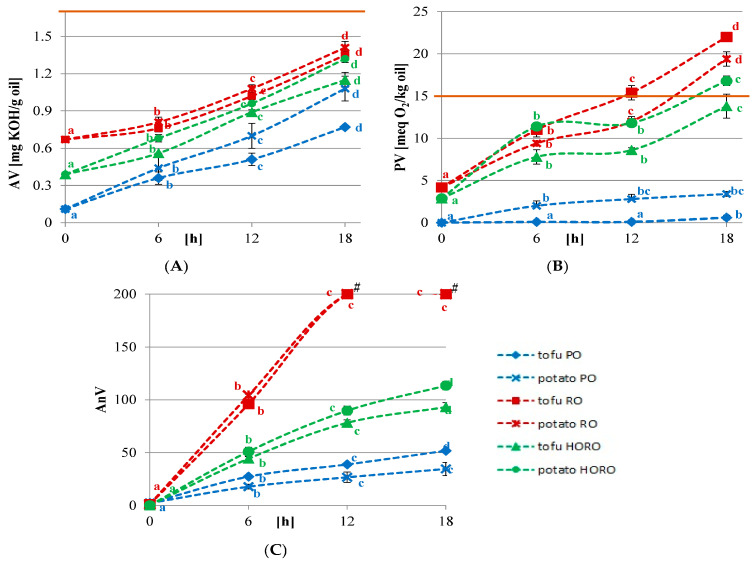
Changes of (**A**) acid values [mg KOH/g oil]; (**B**) peroxide values [meq O_2_/kg oil]; (**C**) *p*-anisidine values of tested oils during deep frying of tofu or potatoes. PO—palm olein; RO—cold pressed rapeseed oil; HORO—cold pressed high oleic rapeseed oil. # *p*-anisidine values above limit of quantification (>200). The values for the specific oil and type of fried product with different letters (a, b, c, d) are significantly different (*p* < 0.05).

The acid values (AVs) of all the tested oils increased slightly during the frying process. After 18 h of deep frying, the oils did not exceed the value of 1.5 mg KOH/g oil (Figure 1A). Based on the Codex Alimentarius, the limit of refined oils is 0.6 and, for cold pressed oils, 4 mg KOH/g oil; thus, before frying all the tested oils were below the limit [29]. The low AVs prove the low amount of free fatty acids of the tested oils. A slight increase of the values is related to some hydrolytic changes, but this process had no significant impact on the quality of the tested oils. The AVs of the oils after the frying process were much below the limit; similar observations were reported also by the other authors [30,31].

The peroxide values (PVs) of rapeseed oils before frying were 2.88 meq O_2_/kg oil, HORO, and 4.19 meq O_2_/kg oil, RO (Figure 1B). This is typical for cold pressed oils, and it has been attributed to the higher hydroperoxides contents of the unrefined oil. Additionally, those oils were pressed from hulled seeds. Removal of the seed coat causes partial damage to the seed’s structure, which leads to the activation of native seed enzymes and to the opening of cells and outflow of oil, and therefore to a rapid deterioration of the quality of the lipid fraction [32]. Still, all the tested oils kept their PVs below the limit (15 meq O_2_/kg oil) [29] during 12 h of frying. After 18 h of deep frying, only the PV of palm olein stayed low (below 4 meq O_2_/kg oil) (Figure 1B). Cold pressed rapeseed oil exceeded the limit of peroxides after 18 h of deep frying. There were some slight differences in the PVs after frying potatoes and tofu. The PVs of PO and HORO increased more after frying starch product compared to protein product. The opposite tendency was noticed for RO. As was already proved in other studies, polyunsaturated fatty acids are readily oxidized to hydroperoxides, thus PO PVs were much lower compared to RO and HORO [30,33,34].

During 18 h of deep frying not only an increase of primary oxidation products was noticed, but also an increase of secondary oxidation products. The longer the heating time, the higher *p*-anisidine values (AnV) of the analyzed oils were observed (Figure 1C). An increase of AnV was the most drastic for RO with values of around 100 after 6 h, and above the limit of quantification after 12 and 18 h of deep frying. A smoother increase was reported for HORO, reaching the values of 113.4 after 18 h of potatoes and 93.08 of tofu deep frying. The AnV of PO after 18 h of deep frying were 34.66 (potatoes) and 51.9 (tofu) (Figure 1C). A significant increase of secondary oxidation products during deep frying was also observed by other authors [35,36]. In other studies, a bigger increase and much higher values of AnV were also noticed after deep frying, compared to the peroxide values (PV) of palm olein and rapeseed oils [35]. For example, after 30 h of deep frying of potatoes at 185 °C, theAnV of the rapeseed oil reached 64.28 [37].

### 3.2. Chlorophyll, Carotenoid Pigments, and Color of Oils

In the studied oils the level of chlorophylls was evaluated because they contribute to the color, and along with metal ions such as Fe or Cu, they increase the susceptibility of oils to oxidative changes [38]. Meanwhile, carotenoids inhibit the oxidative changes by protecting the oil from free radical autoxidation, and they also affect oil color [38].

**Figure 2:**
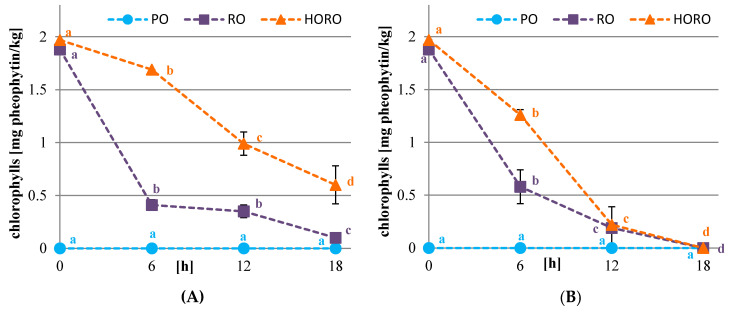
Changes in the content of chlorophyll pigments [mg pheophytin/kg oil] of tested oils during deep frying of potatoes (**A**) or tofu (**B**). PO—palm olein; RO—cold pressed rapeseed oil; HORO—cold pressed high oleic rapeseed oil. The values for the specific oil with different letters (a, b, c, d) are significantly different (*p* < 0.05).

**Figure 3:**
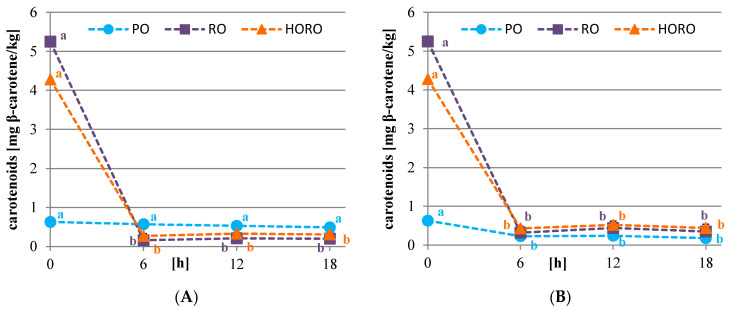
Changes in the content of carotenoid pigments [mg β-carotene/kg oil] of tested oils during deep frying of potatoes (**A**) or tofu (**B**). PO—palm olein; RO—cold pressed rapeseed oil; HORO—cold pressed high oleic rapeseed oil. The values for the specific oil with different letters (a, b) are significantly different (*p* < 0.05).

The content of chlorophylls, especially in cold pressed oils, depends on the amount of this pigment in the seeds and their maturity level. The more mature the seeds are, the less chlorophyll is in the oil [39], which is beneficial due to its oxidative stability [39]. The study showed that palm olein did not contain chlorophyll pigments and had a very low content of carotenoids (0.63 mg β-carotene/kg) due to fractionation and refining processes. Cold pressed RO and HORO were characterized by a similar content of chlorophylls (1.88 and 1.97 mg pheophytin/kg, respectively) (Figure 2), but a different content of total carotenoids (5.25 and 4.28 mg β-carotene/kg, respectively) (Figure 3). PO, RO, and HORO oils, in comparison to the literature data [40,41], had a low concentration of chlorophylls, which was advisable in view of the intended use for frying. However, the tested oils had an average content of carotenoids, about half as much as the oils analyzed by Symoniuk et al. [40], and several times higher than the oils studied by Redondo-Cuevas et al. [42].

The frying process has significantly affected the pigment content of oils. It was observed that the degradation of chlorophylls was significantly lower in the oils after potatoes frying than in the oils after tofu frying. Furthermore, chlorophylls were more stable when frying the starchy material in HORO than in RO. The highest amount of these substances after 18 h of treatment remained in HORO oil (0.6 mg pheophytin/kg) (Figure 2A). The dynamics of chlorophyll degradation were much faster when tofu, a protein-based material, was fried. After 12 h of treatment, a negligible amount of these substances remained in cold pressed rapeseeds oils (0.22 mg pheophytin/kg), and after 18 h they were not detected at all (Figure 2B). Carotenoids present in oils were significantly less stable than chlorophylls. After 6 h of frying in RO and HORO, both potatoes or tofu, the total carotenoid content was reduced to very low levels (0.16–0.43 mg/kg), and remained at that amount until the end of frying (Figure 3A,B). A slight increase in the values spectrophotometrically determined was observed after 12 h of frying, which could be caused by the degradation of chlorophyll pigments to brown compounds. In PO, the carotenoid pigments were more stable than those in cold pressed rapeseed type oils, due to the different chemical composition. The carotenoid content decreased slightly throughout the 18 h potato frying process, while tofu frying resulted in a significant decrease in content after 6 h which remained stable to the end of deep frying.

**Figure 4:**
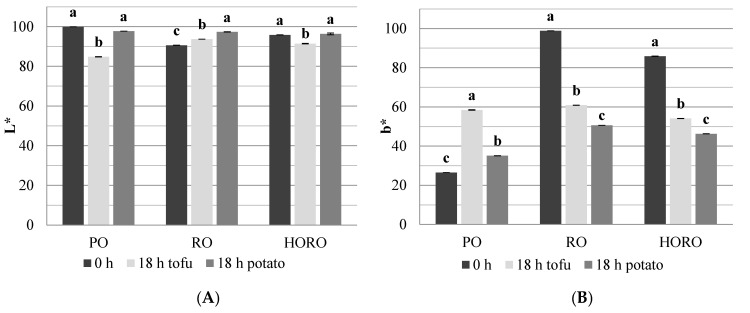
Changes in the color values L* (**A**) and b* (**B**) of tested oils during deep frying of potatoes or tofu. PO—palm olein; RO—cold pressed rapeseed oil; HORO—cold pressed high oleic rapeseed oil. The values for the specific oil with different letters (a, b, c) are significantly different (*p* < 0.05).

Carotenoids, like chlorophylls, affect the color of oil, which in refined oils is subjected to adjustment to meet consumer preferences. The fresh oils showed a color characteristic of the respective type. All studied oils had high L* values, which means that they were bright and transparent (Figure 4A). RO and HORO had lower L* values because of a much higher pigments content. This was also reflected in the high b* values of these oils.

Deep frying affected the oils color parameter b* more drastically than parameter L*. After 18 h of tofu frying, PO and HORO were significantly darker than after potato frying. Only for RO, the frying process brightened it up regardless of the fried material. HORO was brighter than PO after 18 h of deep frying, because it is a characteristic feature of high oleic oils that they are brighter in color than palm fats after heat treatment [43].

The b* value is related to the content of yellow pigments like carotenoids. In the case of cold pressed RO and HORO, the b* value decreased in the same way because of carotenoids degradation. A significantly lower b* value was obtained for the oils in which potatoes were fried (Figure 4B). The PO which originally contained very little carotenoids had a very low b* value in comparison to RO and HORO. During the deep frying of tofu or potatoes, this value increased significantly due to the formation of oxidative substances in the oils and Maillard compounds in the fried raw materials that were transferred to the oils. In view of the above, oil darkening and pigment loss are great indicators of the thermo-oxidative changes taking place in the oils.

### 3.3. Smoke Point

**Figure 5:**
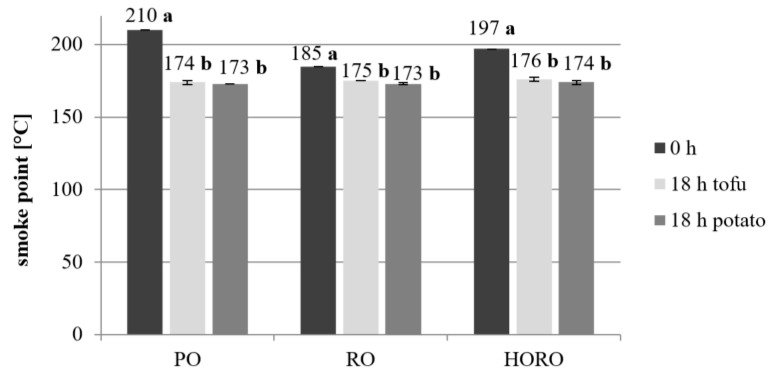
Changes in smoke point [°C] of tested oils after frying of tofu or potatoes. PO—palm olein; RO—cold pressed rapeseed oil; HORO—cold pressed high oleic rapeseed oil. The values for the specific oil and type of fried product with different letters (a, b) are significantly different (*p* < 0.05).

The smoke point of the oils before frying already indicates that the most suitable for frying is PO (210 °C), followed by HORO (197 °C), and RO (185 °C) is the least suitable (Figure 5). The initial smoke points of all the oils were higher than the frying temperature used in the study (180 °C). Such a high smoke point of palm olein was also observed by other authors reaching between 210 °C and 220 °C [31,35].

An interesting result is that, after 18 h of deep frying, the smoke point decreased to the same level of around 173–175 °C for each oil after frying both products. A significant decrease of the smoke point after frying was also reported by other authors, reaching 177.5 °C after 5 days of deep frying [44]. Similar results were obtained by Haizam et al. [45]—the smoke point of palm olein was 212 °C, while after 16 h of frying potatoes it was 190 °C. In the case of HORO, the smoke point dropped from 197 °C to 174 °C (potatoes) and 176 °C (tofu). Similar results were reported by Matthäus [3]: the smoke point of fresh high oleic rapeseed oil was 194 °C, and after deep frying of potatoes for 18 h, this value dropped to 175 °C. In the case of RO, the smoke point dropped from 185 °C to around 174 °C after deep frying. The smoke point in oils during the frying process should not decrease by more than 50 °C [46], and in our study this was not observed in any of the tested oils. The type of fried product did not have a significant effect on the final value of the smoke point of individual oils.

### 3.4. FAMEs

Fatty acids composition has a significant effect on oxidative stability, as well on nutritional value. Unfortunately, the fatty acids composition recommended from a nutritional point of view does not match the best oxidative stability of these fats [35]. The predominant fatty acids of palm olein were palmitic (C16:0—41.35%), oleic (C18:1—41.41%), linoleic (C18:2—10.67%) and stearic (C18:0—4.01%) acids (Table 1). Such a high content of saturated and monounsaturated fatty acids makes this oil less prone to the oxidation processes. It was reported that higher levels of oleic acid (42–63%) and lower level of linoleic acid (23–37%) would result in an increased stability of the oils. Thus, the reduced level of C18:2 and increased level of C18:1 in HORO were expected to increase the oxidative stability of this oil compared to RO. A clearly dominating fatty acid of rapeseed oils was oleic acid (RO—63.60%; HORO—76.96%). The total content of PUFA of cold pressed rapeseed oil was much higher compared to other tested oils, reaching 26.77%.

It was found that during the frying process the content of PUFAs significantly decreased in RO (21.68% after 18 h of tofu deep frying) and in HORO (10.90% after 18 h of tofu deep frying). A slight decrease of the content of fatty acids was observed both for polyunsaturated (C18:3, C18:2) and monounsaturated (C18:1) fatty acids. On the other hand, saturated fatty acids (C12:0, C14:0, C16:0, C18:0, C20:0) did not decrease after 18 h of potatoes and tofu deep frying (Table 1). The same observation was reported for HORO by Xu et al. [47]. A significant oxidation of unsaturated fatty acid, which converts mostly to primary and secondary oxidation products during the frying process, was also reported by other authors [44,48]. The differences in FFA formation with frying can be related to the physicochemical characteristics of the individual oils and their response to moisture and heating during frying.

### 3.5. Rancimat Test

As was found in other studies, Rancimat test is more useful for testing the oxidation of fats after frying, compared to the peroxide values [18,31,34]. Hydroperoxides degrade quickly and it is difficult to measure them in the chemical analysis of PV. However, Rancimat test can measure more precisely and the level of degradation changes can be measured and observed based on the induction period [30,34]. In this study, during the deep-frying process, the oxidation stability index (OSI) was shorter after 6, 12, and 18 h for each tested oil (Table 2). The OSI was significantly different for each kind of tested oil, the shortest for the RO and the longest for the PO; thus, based on this parameter the RO should not be applied for frying. On the other hand, even though the initial OSI of the HORO was relatively high (10.26 h), it decreased very much (1.02 h—tofu; 0.72 h—potatoes) after 18 h of deep frying at 180 °C. Only the OSI of the PO after 18 h of deep frying was still relatively high (6.80 h—tofu; 7.76 h—potatoes) (Table 2). Other studies reported a slightly higher OSI of fresh rapeseed oil (10.2–14.1), but the tested oil was refined [34,40]. Ismail [31] reported an even much higher OSI of fresh palm olein (21 h) but the main parameter of the test was slightly different (a temperature of 110 °C). Thus, it should not be surprising that several million tons of palm oil and palm olein are used annually in the world for frying.

### 3.6. Total Polar Compounds

**Figure 6:**
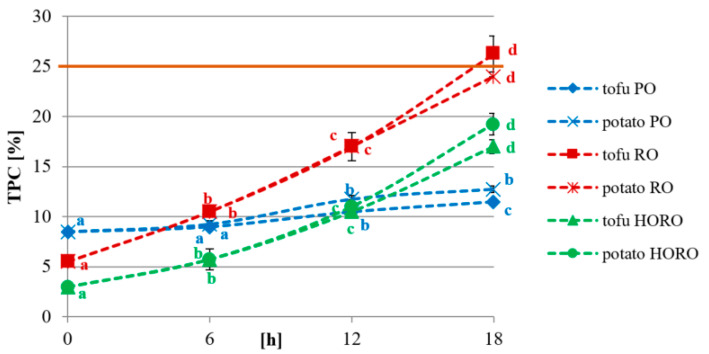
Changes in total polar compounds [%] of tested oils during frying of tofu or potatoes. PO—palm olein; RO—cold pressed rapeseed oil; HORO—cold pressed high oleic rapeseed oil. The values for the specific oil and type of fried product with different letters (a, b, c, d) are significantly different (*p* < 0.05).

The amount of total polar compounds (TPC) is an important factor to assess the oil quality and it is frequently used in the industry. TPC include mainly monoglycerides, diglycerides and free fatty acids, naturally present in oils or formed during heating such as the frying process. In the present study, the amount of total polar compounds significantly increased during frying. The increase was the smallest in palm olein, starting from 8.5% before frying and reaching 11.5% after 18 h of tofu frying and 12.75% after 18 h of potato frying (Figure 6). Ismail [31] reported 5% of total polar compounds of fresh palm olein, which increased up to 11% after potato frying. A much larger increase of TPC was observed in RO and HORO. The values differ because of the different content of TPC in the oils before heating, but the tendency was similar (Figure 6). The initial TPC of HORO was 3% and reached above 17% after 18 h of heating, while RO had an initial TPC 5.5% and increased above 24% after 18 h of heating at 180 °C (Figure 6). The study of sunflower and high oleic sunflower oils also reported a significant increase of TPC: from 1.76% to 15.93% after 24 h of deep frying at 180 °C [49]. In addition, Kmiecik et al. [50] reported that the final level of TPC in partially hydrogenated rapeseed oil used for frying the traditional and fast French fries was 6.3 and 5.3 time higher compared to fresh oil, respectively. The limit of TPC in oils differs depending on the country, e.g., Poland, Italy, Spain, France, and India have set a limit of 25%, Germany, of 24%, while Austria, Netherlands, Australia, China, and Switzerland have set it at 27% [35]. In this study, only RO after 18 h of deep frying was close to exceed the upper limit. All the tested samples had values below the limit.

### 3.7. Oxidizability (COX) Value and Nutritional Quality Indices of Oils

As was mentioned previously, the selection of a frying medium is very important, since a high amount of the oil is absorbed during the process, in some cases reaching 40% of the total food product weight [9]. Therefore, it seems expedient to investigate the effect of frying on the nutritional quality indices of oils. The studies done by many researchers on the effect of fatty acids on cardiovascular and heart diseases gave the opportunity to determine lipid quality indices by Ulbricht and Southgate [26], as well as Santos-Silva et al. [27]. These indices characterize the conclusions reached by the mentioned researchers in their studies. One of the conclusions was the observation that not all saturated acids (SFA) affect the elevation of LDL cholesterol in blood in the same way. The fatty acid composition obtained in this study allowed the calculation of indices like COX value, AI index, TI index, and hH ratio.

COX value is a beneficial element usually taken as an evaluation of the oil’s tendency to undergo autoxidation. Based on the calculated COX values, the oxidative stabilities of the three tested oils were in the following order: PO > HORO > RO (Table 3). This order corresponds to the results of Rancimat test. Among all the analyzed oils, RO was the most susceptible to oxidation because of the highest content of C18:2 and C18:3 fatty acids, which, according to Fatemi and Hammod [25], have the highest relative rates of oxidation. It should be noted that HORO had a COX value twice better as that of RO. Comparing with the literature, PO had a typical COX value for palm oils declared for frying [51]. Regarding the results of the oils after 18 h of deep frying, the COX values decreased statistically in a significant manner, regardless of the fried material. This means that oxidative changes took place in the oils, with the highest intensity in RO. However, it should be considered that in the evaluation of the oxidative stability of oils, in addition to COX values, also the content of tocopherols and other substances of antioxidant character is of key importance [52,53].

Both (RO and HORO) oils were characterized by a lower value of the AI index than palm olein, both before and after deep frying showed approximately a 20-fold higher value of this index than PO. These results indicate that both cold pressed rapeseed oils had very low atherosclerotic potential, while palm olein had it very high; thus, in terms of atherosclerosis prevention, it is not recommended for frying.

Similar to the AI index, also in the case of the TI index PO took higher values than RO and HORO, both before and after 18 h of the deep frying process (Table 3). Similarly to the AI index, the TI index values did not change in the particular oils during the frying of potatoes or tofu. Based on the calculations, palm olein was found to have a greater prothrombotic indice than different types of rapeseeds oils and, in this regard, it is not recommended for frying.

The hH ratio differed drastically between PO and rapeseed oils. HORO had the best ratio; that is why it is the best choice for frying in terms of blood cholesterol levels. However, during deep frying HORO had the highest decreases of hH ratio. The hH ratio for RO decreased less significantly after deep frying, and for PO heat treatment caused no change.

It seems that the type of fried material did not have much influence on the changes in the fatty acid profile of the tested oils.

### 3.8. PCA Analysis of Oil Samples

**Figure 7:**
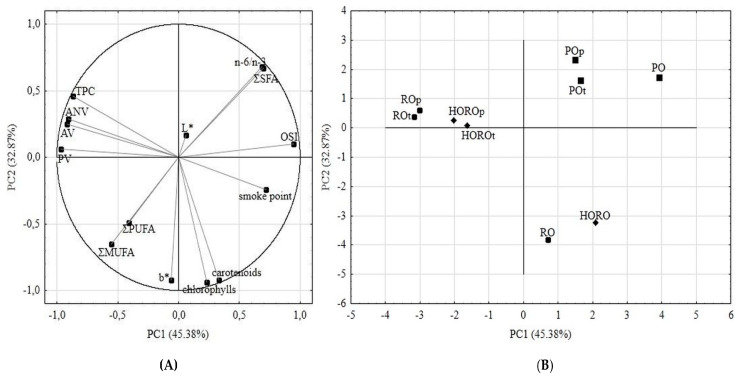
PCA analyses results. (**A**) Score plot, PC1 versus PC2 of all samples; (**B**) Score plot, PC1 versus PC2 of data from determinations used as variables. PO—palm olein; RO—cold-pressed rapeseed oil; HORO—high-oleic cold-pressed rapeseed oil; the subscripts t and *p* indicate oil samples after 18 h of frying tofu or potatoes, respectively.

In order to comprehensively demonstrate the alterations occurring in the types of tested oils after 18 h of deep frying of starch- or protein-based food, a chemometric analysis in the form of a PCA analysis was conducted on the qualified gathered data. In the PCA analysis, the eight principal components were created, out of which the first two explained 78.25% of the total variability. Figure 7B shows the distribution of samples in two-dimensional space in relation to the first and second principal components, based on correlations and loadings of uploaded data of classified analyses (Figure 7A).

Based on the PCA analysis (Figure 7A,B), it can be concluded that heat processing changed the determined parameters in PO the least, followed by HORO, whereas frying with RO affected its quality the most. For PO, the least alterations occurred in the oxidation stability index (OSI), ΣSFA and n − 6/n − 3 ratio, while the most changes concerned smoke point and the PV, AV and AnV values. The dynamics of the thermo-oxidative modifications of the composition of all the tested oils were best represented by the PV, AV and AnV values, together with the TPC percentage. The PCA analysis also showed high binding of color value b* with carotenoid and chlorophylls pigments content. The samples of RO and HORO as rapeseed matrixes, both before and after deep frying, were placed close together on a score plot. However, chemometric analysis indicated a light composition difference of HORO, which was maintained during the frying process. For RO and HORO, the most characteristic changes concerned color b* value, carotenoids and chlorophylls pigments, ΣMUFA, ΣPUFA.

According to PCA statistics, the type of fried product had little effect on the quality of the oils after 18 h of heat treatment. The greatest effect was noted for PO. The conclusion from that is that the most important indicator in terms of frying is the original composition of the oil, i.e., its fatty acid composition and the content of bioactive compounds promoting or inhibiting oxidative changes.

## 4. Conclusions

Extreme conditions such as high temperature (180 °C), water (average water content of potatoes and tofu is 80%) and air access over a long time (18 h) lead to notable physical and chemical changes in the tested oils. It was concluded that after 18 h of deep frying of both the starch and protein product at a temperature of 180 °C, the greatest degradation changes occurred in cold pressed rapeseed oil. High oleic rapeseed oil was a bit more stable during the thermal treatment, but it is still not recommended for deep frying purposes. Palm olein was the most resistant to thermo-oxidative changes during deep frying, however, in terms of nutritional quality, it turned out to be the least valuable. The high stability of palm olein is due to the high content of saturated fatty acids compared to rapeseed oils.

Additionally, it was observed that the type of fried product had an effect on some quality indicators of the oils after 18 h of deep frying. Frying of starch product like potato affect oil quality deterioration more (higher AV and PV) than frying protein product. The greatest decrease in quality during potato frying was noted for HORO. The opposite situation was in terms of chlorophylls content—frying the tofu degraded these pigments to zero. The b* value which is related to the content of carotenoids was significantly lower obtained for oils in which potatoes were fried. The type of fried product had no effect on OSI, fatty acid composition, and nutritional indices.

## Figures and Tables

**Table 1:** Content of fatty acids [g/100 g] in tested oils before and after of 18 h deep frying of potatoes or tofu.

	PO			RO			HORO		
	Before Frying	After Frying Potatoes	After Frying Tofu	Before Frying	After Frying Potatoes	After Frying Tofu	Before Frying	After Frying Potatoes	After Frying Tofu
**C12:0**	0.58 ± 0.04	0.59 ± 0.06	0.59 ± 0.06	nd	nd	nd	nd	nd	nd
**C14:0**	0.97 ± 0.07	1.00 ± 0.02	1.01 ± 0.04	nd	nd	nd	nd	nd	nd
**C16:0**	41.35 ± 0.14	41.45 ± 0.51	41.45 ± 0.51	4.13 ± 0.02	4.14 ± 0.04	4.13 ± 0.02	3.01 ± 0.04	3.19 ± 0.08	3.21 ± 0.05
**C16:1**	0.20 ± 0.00	0.19 ± 0.01	0.19 ± 0.00	0.21 ± 0.01	0.20 ± 0.01	0.21 ± 0.01	0.20 ± 0.01	0.21 ± 0.00	0.20 ± 0.00
**C18:0**	4.01 ± 0.00	4.03 ± 0.02	4.00 ± 0.00	1.82 ± 0.01	1.88 ± 0.08	1.90 ± 0.04	1.99 ± 0.01	2.02 ± 0.01	1.99 ± 0.01
**C18:1**	41.41 ± 0.14	41.39 ± 0.23	41.29 ± 0.28	63.60 ± 0.14	61.80 ± 0.72	62.55 ± 0.83	76.96 ± 1.03	75.25 ± 1.23	75.71 ± 1.94
**C18:2**	10.67 ± 0.14 ^a^	9.87 ± 0.01 ^b^	9.83 ± 0.05 ^b^	18.21 ± 0.11 ^a^	15.68 ± 0.73 ^b^	15.52 ± 0.66 ^b^	8.71 ± 0.11 ^a^	7.90 ± 0.02 ^b^	8.60 ± 0.08 ^a^
**C18:3**	0.23 ± 0.00	0.20 ± 0.00	0.21 ± 0.01	8.56 ± 0.07 ^a^	6.21 ± 0.16 ^b^	6.16 ± 0.45 ^b^	2.92 ± 0.11 ^a^	1.72 ± 0.07 ^b^	2.31 ± 0.30 ^a^
**C20:0**	0.37 ± 0.01 ^a^	0.42 ± 0.00 ^b^	0.44 ± 0.03 ^b^	0.55 ± 0.01	0.46 ± 0.04	0.51 ± 0.07	0.66 ± 0.02	0.60 ± 0.04	0.62 ± 0.01
**C20:1**	nd	nd	nd	1.28 ± 0.04	1.27 ± 0.01	1.27 ± 0.03	1.28 ± 0.02 ^a^	1.30 ± 0.02 ^a^	1.00 ± 0.19 ^b^
**C22:0**	nd	nd	nd	0.27 ± 0.04	0.26 ± 0.06	0.29 ± 0.01	0.58 ± 0.08 ^a^	0.32 ± 0.04 ^b^	0.27 ± 0.04 ^b^
**Σ SFA**	47.27 ± 0.03	47.48 ± 0.45	47.49 ± 0.45	6.76 ± 0.06	6.73 ± 0.06	6.82 ± 0.01	6.24 ± 0.01	6.12 ± 0.14	6.19 ± 0.07
**Σ MUFA**	41.61 ± 0.14	41.58 ± 0.23	41.48 ± 0.28	65.09 ± 0.19 ^a^	63.27 ± 0.72 ^b^	65.06 ± 0.81 ^a^	78.43 ± 1.06	76.76 ± 1.25	76.90 ± 2.14
**Σ PUFA**	10.90 ± 0.14 ^a^	10.07 ± 0.01 ^b^	10.03 ± 0.06 ^b^	26.77 ± 0.18 ^a^	21.88 ± 0.89 ^b^	21.68 ± 1.12 ^b^	11.62 ± 0.00 ^a^	9.62 ± 0.09 ^c^	10.90 ± 0.37 ^b^
**n − 6/n − 3**	46.39 ± 0.61 ^b^	49.33 ± 0.04 ^a^	47.95 ± 1.41 ^ab^	2.13 ± 0.01 ^b^	2.53 ± 0.05 ^a^	2.52 ± 0.08 ^a^	2.99 ± 0.15 ^c^	4.59 ± 0.18 ^a^	3.75 ± 0.45 ^b^

PO—palm olein; RO—cold-pressed rapeseed oil; HORO—high-oleic cold-pressed rapeseed oil. Σ SFA—total saturated fatty acids; Σ MUFA—total monounsaturated fatty acids; Σ PUFA—total polyunsaturated fatty acids; n − 6/n − 3—ratio of omega-6 to omega-3 fatty acids; nd—not detected. The values represent means ± standard deviation (*n* = 3). The values in a row for the specific oil with different letters (a, b, c) are significantly different (*p* < 0.05). The values without letters are not significantly different (*p* < 0.05).

**Table 2:** Changes in the oxidation stability index (OSI) [h] in the Rancimat test of oils.

Type of Fried Product	Time of Frying [h]	PO	RO	HORO
potatoes	0	13.05 ± 0.10 ^a^	3.22 ± 0.11 ^a^	10.26 ± 0.22 ^a^
	6	10.95 ± 0.27 ^b^	2.03 ± 0.26 ^b^	7.16 ± 0.27 ^b^
	12	10.09 ± 0.41 ^b^	1.37 ± 0.05 ^c^	1.30 ± 0.07 ^c^
	18	7.76 ± 0.31 ^c^	0.73 ± 0.07 ^d^	0.72 ± 0.23 ^d^
tofu	0	13.05 ± 0.10 ^a^	3.22 ± 0.11 ^a^	10.26 ± 0.22 ^a^
	6	11.23 ± 0.33 ^b^	2.59 ± 0.25 ^b^	7.22 ± 0.18 ^b^
	12	9.61 ± 0.29 ^c^	1.03 ± 0.09 ^c^	2.39 ± 0.25 ^c^
	18	6.80 ± 0.39 ^d^	0.90 ± 0.29 ^c^	1.02 ± 0.38 ^d^

RO—cold-pressed rapeseed oil; HORO—high-oleic cold-pressed rapeseed oil; PO—palm olein. The values represent means ± standard deviation (*n* = 3). The values in a column for individual type of fried product with different letters (a, b, c, d) are significantly different (*p* < 0.05).

**Table 3:** Calculated oxidizability value and nutritional quality indexes of tested oils before and after of 18 h deep frying of potatoes or tofu.

	PO			RO			HORO		
	Before Frying	After Frying Potatoes	After Frying Tofu	Before Frying	After Frying Potatoes	After Frying Tofu	Before Frying	After Frying Potatoes	After Frying Tofu
**COX**	1.56 ± 0.01 ^a^	1.47 ± 0.00 ^b^	1.47 ± 0.01 ^b^	4.36 ± 0.03 ^a^	3.57 ± 0.12 ^b^	3.56 ± 0.16 ^b^	2.30 ± 0.00 ^a^	1.94 ± 0.02 ^b^	2.14 ± 0.09 ^ab^
**AI**	0.87 ± 0.00	0.89 ± 0.00	0.89 ± 0.01	0.04 ± 0.00	0.05 ± 0.00	0.05 ± 0.00	0.03 ± 0.00	0.04 ± 0.00	0.04 ± 0.00
**TI**	1.73 ± 0.00	1.76 ± 0.01	1.77 ± 0.03	0.09 ± 0.00	0.10 ± 0.00	0.10 ± 0.00	0.10 ± 0.00	0.11 ± 0.00	0.11 ± 0.00
**hH**	1.24 ± 0.00	1.21 ± 0.01	1.21 ± 0.02	21.91 ± 0.03 ^a^	20.24 ± 0.56 ^b^	20.66 ± 0.04 ^ab^	29.43 ± 0.76 ^a^	26.61 ± 0.67 ^b^	26.20 ± 0.53 ^b^

PO—palm olein; RO—cold-pressed rapeseed oil; HORO—high-oleic cold-pressed rapeseed oil. COX—calculated oxidizability value; AI—atherogenic index; TI—thrombogenic index; hH—the ratio of hypocholesterolemic to hypercholesterolemic fatty acids. The values represent means ± standard deviation (*n* = 3). The values in a row for the specific oil with different letters (a, b) are significantly different (*p* < 0.05). The values without letters are not significantly different (*p* < 0.05).

## Data Availability

All data created and analyzed during the experiments was presented in this study.
